# Prevalence of common mental health problems and associated factors among university students visiting Supara mental health service: A cross-sectional study

**DOI:** 10.12688/f1000research.126054.4

**Published:** 2025-03-14

**Authors:** Pantri Kirdchok, Varuna Kolkijkovin, Wanida Munsukpol, Chotiman Chinvararak

**Affiliations:** 1 Department of Psychiatry, Faculty of Medicine Vajira Hospital, Navamindradhiraj University, Bangkok, 10300, Thailand

**Keywords:** Mental health problems, University students, Mental health service, Thailand

## Abstract

**Background:** Early studies found that the mental health problems rate was relatively high in university students. We aimed to investigate the prevalence of mental problems and associated factors in university students.

**Methods:** We conducted a cross-sectional descriptive study at Supara mental health service in the Faculty of Medicine Vajira Hospital between February 2020 to June 2021. The primary outcome was the prevalence of psychiatric diagnosis according to the 10th revision of the International Statistical Classification of Diseases and Related Health Problems (ICD-10). The secondary assessments included the Patient Health Questionnaire-9 (PHQ-9), 8 items from the Mini International Neuropsychiatric Interview (MINI) to assess suicidal risk (8Q), , and the Thai Mental Health Indicator (TMHI-15). The prevalence of mental health problems was presented by frequency and percentage. In addition, multivariable regression analysis was used to identify potential predictors of mental health problems.

**Results:** A total of 184 participants (62% female; mean age = 22.49 years (SD 3.93) were recruited. The depressive disorders, adjustment disorders, and anxiety disorders rates were 57.1%, 15.2% and 13.6%, respectively. Grade point averages (GPAs) below 3.0 (OR=3.09, 95%CI: 1.17-8.14) and a family history of mental disorder (OR=3.40, 95%CI: 1.10-10.48) were significant associated factors of moderate to severe mental health problems. Detecting and screening these factors may help the university to provide early detection and treatment for students.

**Conclusions:** Depressive disorders were the most common mental health disorders. Females, low GPAs and a family history of mental disorder were predictors of moderate to severe mental health problems.

## Introduction

Mental health issues are one of the leading global problems among university students [ Macaskill, 2013; Akhtar *et al.*, 2020]. Early studies found that mental health problems, especially in medical and nursing students [ MacLean *et al.*, 2016, Tung *et al.*, 2018], disturbed not only the quality of life but also affected academic performance [ Bakker *et al.*, 2020] and empathic feelings for patients [ Lins *et al.*, 2015]. In addition, examples of common mental health problems in university students are stress [ Pacheco *et al.*, 2017], burnout [ Pacheco *et al.*, 2008], sleep problems [ Azad *et al.*, 2015; Chinvararak *et al.*, 2021], depression [ Tung *et al.*, 2018, Pacheco *et al.*, 2017], anxiety [ MacLean *et al.*, 2016; Pacheco *et al.*, 2017], and suicidal ideation [ Pacheco *et al.*, 2008]. Prior studies showed that the prevalence of depression in medical students globally and in Thailand ranged between 2-30% [ Rotenstein *et al.*, 2016; Phomprasith *et al.*, 2022], while the anxiety rate was approximately 6-78% [ Puthran *et al.*, 2016]. Other psychiatric disorders, such as obsessive-compulsive disorder (OCD) and substance use disorder, were also found [ Pacheco *et al.*, 2017]. However, most of the results came from a community-based study. There is still limited data on mental health problems in a clinical-based study in which clients decided to consult mental health services.

Furthermore, factors found to be associated with mental health problems in medical students include course year, academic pressure, relationship problems, bullying, sleep deficiency, high workload, and chronic medical diseases [ Puthran *et al.*, 2016; Firth, 1986]. However, there are several reports on the prevalence of mental issues; the results revealed that most medical students are more likely not to seek mental health help [ Quince *et al.*, 2012]. This cause may result from a negative attitude toward psychiatric disorders, fear of stigmatisation, or confidentiality concern about the treatment process [ Jacob *et al.*, 2020; Hankir *et al.*, 2014].

**Figure 1.**
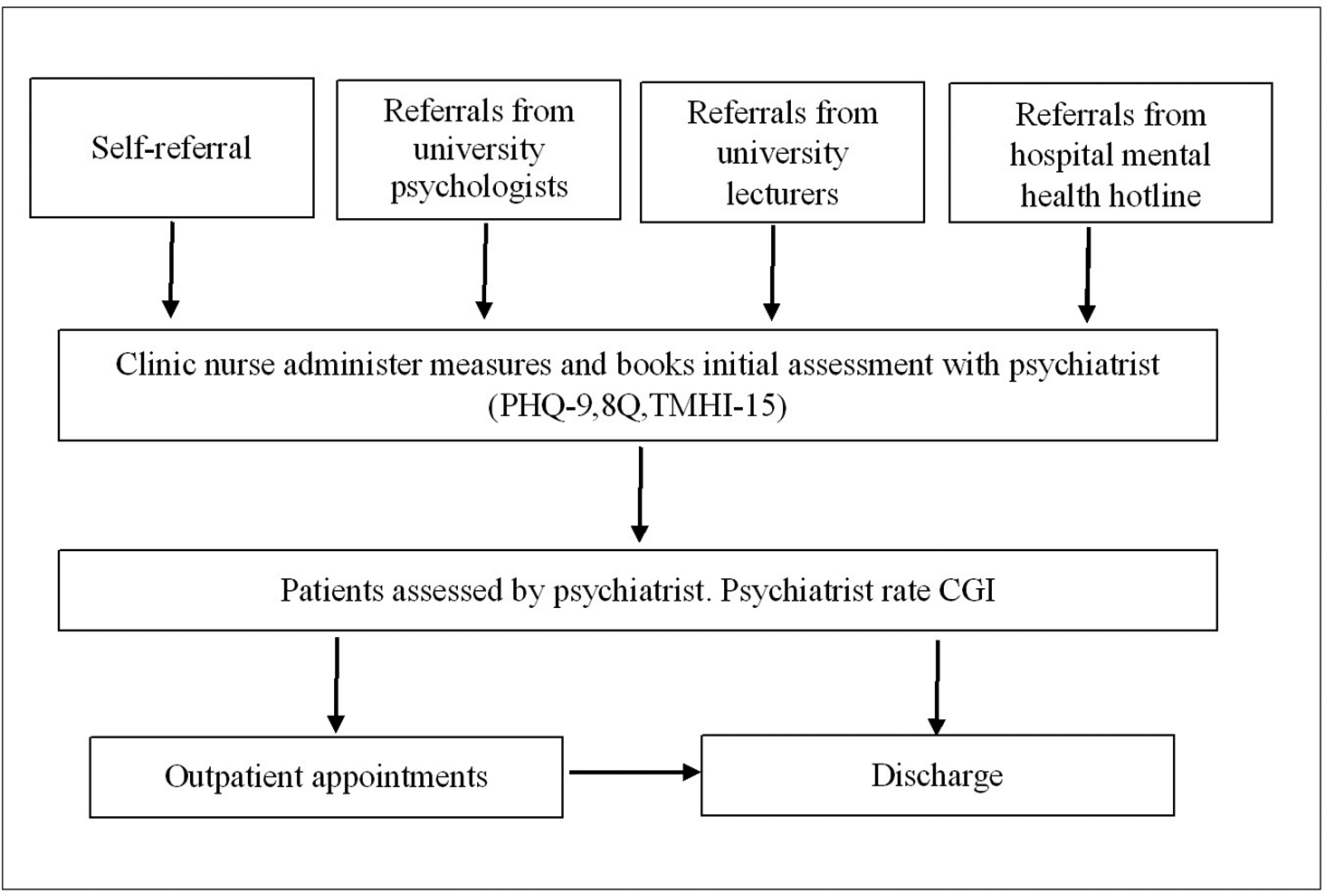
Supara mental health service process map.

“Supara” mental health clinic is a specialised in-house clinic that provides mental health service for students and staff of Navamindradhiraj University. This clinic was established and implemented in 2020 by the department of psychiatry, Faculty of Medicine Vajira Hospital, which is affiliated with Navamindradhiraj University. The clinic intended to increase access to mental health services for university students by addressing confidentiality, privacy, and waiting time. There are psychologists, mental health nurses, and psychiatrists who provide comprehensive mental health evaluation and treatment ( Figure 1).

Although much research has studied the prevalence and associated factors of mental health problems in students, Supara mental health clinic is a specific service model. Consequently, this study aimed to investigate the prevalence of common mental health problems among undergraduate and postgraduate students of Navamindradhiraj University who decide to seek the mental health service. The results of this study will be used to plan for further service improvements based on the prevalence of common problems and related factors.

## Methods

### Ethics and consent

We obtained approval from the Ethical Committee of the Institutional Review Board of the Faculty of Medicine Vajira Hospital on June 26 ^th^, 2021 (COA no. 111/2564). All participants were asked to provide written informed consent to complete routine patient-reported outcome measures (PROMs) when using the clinic’s service. Informed consent described the purpose of data collection, i.e. to assess treatment effectiveness in the routine clinic. They were also informed that their information might be used in the future under the supervision of the ethical committee. The data used in this study were retrospectively retrieved from the clinic’s records; participants were not directly involved.

### Study design, setting, and participants

We conducted a cross-sectional descriptive study based on STROBE guidelines [ von Elm *et al.*, 2008]. We retrospectively investigated the mental health record form from Supara mental health service's database. The sample size was calculated according to the Cochrane formula [ Wayne, 1995]. The number of students visiting (N) Supara mental health service between February 2020 to June 2021 was 260. The sample size was estimated by p = 0.196 according to the study by Limsricharoen *et al.* [2014]. Using alpha at 0.05 and error (d) at 0.05, the required sample size was 140.

### Inclusion/exclusion criteria

Participants were included if they: (a) were a student of Navamindradhiraj university; (b) were aged 18 years and older; (c) had a record of using the mental health service. Participants were excluded if their record data was not sufficient to be analysed.

### Data collection

#### Materials

Participants visiting Supara mental health service would perform the questionnaires in-person on the first visit. We explored the pre-existing database and created the data record form. The data record form comprised five sections: 1) demographic data including sex, age, admission route to the university, course year, faculty, department, underlying disease, waiting time before receiving the first evaluation, satisfactory scores which is a Likert-type scale from 0 to 10 (0 = poor, 10 = excellent); 2) clinical characteristics including diagnosis recorded according to the 10th revision of the International Statistical Classification of Diseases and Related Health Problems (ICD-10) [ World Health Organization, 1992], from a consultant psychiatrist, number of visits to the mental health service, a history of psychiatric treatment and, prescription of psychotropic medications; 3) Clinical Global Impression Scale (CGI); 4) Patient Health Questionnaire-9 (PHQ-9); 5) 8 items from the Mini International Neuropsychiatric Interview (MINI) to assess suicidal risk (8Q); and 6) Thai Mental Health Indicator (TMHI-15).

CGI was used to assess the severity of the illness. It is divided into 7 levels of severity, sorted into ascending severity scores: 1) normal or not at all ill, 2) borderline mentally ill, 3) mildly ill, 4) moderately ill, 5) markedly ill, 6) severely ill, and 7) among the most extremely ill patients [ Busner and Targum, 2007].

PHQ-9 Thai version consists of a total of 9 questions. The total score of PHQ-9 is categorised into 4 levels of severity of depression: 1) normal (0–6), 2) mild (7–12), 3) moderate (13–18), and 4) severe (≥19). The sensitivity and specificity of PHQ-9 to distinguish depression are 84% and 77%, respectively [ Lotrakul *et al.*, 2008].

8Q was developed to measure suicidal risk by Kittirattanapaiboon following the MINI. It is classified as suicidal risk into 1) low risk (1-8), 2) moderate risk (9-16), and 3) high risk (>17) [ Kittirattanapaiboon and Khamwongpin, 2005].

TMHI-15 short version was developed by Monhkol *et al.* and aimed to measure the mental health of Thai people. The total scores can be divided into 3 groups: 1) better than average mental health (51-60), 2) average mental health (44-50), and 3) below average mental health (<43) [ Mongkol *et al.*, 2013].

All questionnaires except CGI in this study were self-rated by participants on their first visit to the Supara mental health service. The CGI scale was rated by psychiatrists on the patient’s first visit. PK and CC extracted the record data independently. If there were any conflicts in the data, the third author would double-check the result and provide the final consensus.

### Statistical analyses

Data were analysed using SPSS software (version 28.0; IBM, Chicago, IL, USA). The prevalence of mental health problems was presented by frequency and percentage. In addition, associated factors to the severity of mental health problems were analysed by the Independent samples t-test, Mann-Whitney U test, Chi-square test or Fisher's exact test, depending on the variable types. Finally, multivariable regression analysis (odds ratio [OR] and 95% confidence interval [CI]) was used to identify potential predictors of mental health problems. We classified the severity of mental health problems by CGI scale: normal to mild (CGI 1-3) and moderate to severe (CGI 4-7). We used the listwise deletion method for handling missing data. P < 0.05 was considered statistically significant.

## Results

Of 184 participants recruited in this study, they had a mean age of 22.49 years old (SD 3.92). Most participants were female (62%), undergraduate (84.8%), and studied in the Doctor of Medicine programme. Most medical students were enrolled at the university through the consortium of Thai Medical Schools admission system (71.4%), and they were preclinical year students (59%). Furthermore, the participants' average grade point average (GPAs) was 3.15 (SD 0.42). The median waiting time for their first psychiatric assessment was 0 days (IQR 0-5). Lastly, the majority of participants were satisfied with the university's mental health service, with a mean score of 9.45 (SD 0.83) ( Table 1).

**Table 1.** Demographic data (n=184).

Variables	n	(%)
Sex		
Male	70	(38.0)
Female	114	(62.0)
Age (years), Mean ± SD	22.49 ± 3.92	
Education level		
Undergraduate	156	(84.8)
Resident or Fellowship	26	(14.1)
Unspecified	2	(1.1)
Faculty of Medicine Vajira Hospital		
Doctor of medicine	105	(57.1)
Bachelor of Science Programme in Paramedic	9	(4.9)
Bachelor of Science Programme in Radiological Technology	6	(3.3)
Resident or Fellowship	25	(13.6)
Kuakarun Faculty of Nursing	17	(9.2)
Faculty of Sciences and Health Technology	15	(8.2)
Institute of Metropolitan Development	2	(1.1)
Urban Community Development	3	(1.6)
Unknown	2	(1.1)
Course year (Doctor of Medicine, n = 105)		
Preclinical year	62	(59.0)
Clinical year	37	(35.2)
Unknown	6	(5.7)
Admission route (Doctor of Medicine, n = 105)		
Bangkok Metropolitan Administration (BMA) School's quota	5	(4.8)
Vajiravudh College's quota	3	(2.9)
First-degree relatives of BMA and university officer's quota	6	(5.7)
Local Government Organization School's quota	1	(1.0)
Others (i.e. special English talent)	2	(1.9)
The consortium of Thai Medical Schools	75	(71.4)
Unknown	13	(12.4)
GPA, Mean ± SD	3.15 ± 0.42	
<3.0	39	(21.2)
≥3.0	99	(53.8)
Unknown	46	(25.0)
Underlying medical disease	37	(20.1)
A family history of mental disorder	26	(14.1)
Waiting time (Median, IQR)	0	(0-5)
Wish to use the telemedicine service	107	(58.2)
Satisfactory scores, Mean ± SD (0 = poor, 10 = excellent)	9.45 ± 0.83	

Table 2 demonstrates the prevalence of psychiatric diagnoses. Among all psychiatric disorders, depressive disorders (57.1%), adjustment disorders (15.2%), and anxiety disorders (13.6%) were the top three most common diagnoses. The median number of visits to the mental health service was 7 (IRQ 3-14). Approximately 86% of participants received psychotropic medications.

**Table 2.** Prevalence of common mental disorders and clinical characteristics.

ICD-10 diagnosis	n	(%)
Depressive disorders	105	(57.1)
Adjustment disorders	28	(15.2)
Anxiety disorders	25	(13.6)
Attention-deficit hyperactivity disorders (ADHD)	16	(8.7)
Insomnia disorder	6	(3.3)
Obsessive-compulsive disorder (OCD)	5	(2.7)
Post-traumatic stress disorder (PTSD)	5	(2.7)
Acute stress reaction	4	(2.2)
Bipolar disorder	2	(1.1)
Mental disorders due to known physiological conditions	1	(0.5)
Eating disorders	1	(0.5)
Somatoform disorder	1	(0.5)
Autistic disorder	1	(0.5)
Tic disorder	1	(0.5)
**Clinical characteristics**		
- Number of visits to the mental health service, Median (IQR)	7	(3-14)
- Psychotropic medications	158	(85.9)
- A history of psychiatric hospitalisation	2	(1.1)

Table 3 reveals the measurement tools used in the mental health service. Around 38% of participants had a CGI scale of 4 (moderately ill). Most participants had mild depression (50%) and low suicidal risk (76.3%) from PHQ-9 and 8Q, respectively. Unsurprisingly, all participants had below-average mental health from TMHI-15.

**Table 3.** PHQ-9, 8Q, TMH-15 and CGI scales.

Measurement scores	n	(%)
CGI scale (n=167)		
Normal, not at all ill	9	(4.9)
Borderline mentally ill	8	(4.3)
Mildly ill	37	(20.1)
Moderately ill	70	(38.0)
Markedly ill	18	(9.8)
Severely ill	9	(4.9)
Among the most extremely ill	3	(1.6)
Unknown	30	(16.3)
PHQ-9 (n = 160)		
- Mild (7–12)	80	(50.0)
- Moderate (13–18)	52	(32.5)
- Severe (≥19)	28	(17.5)
8Q (n = 160)		
- Low risk (0-8)	122	(76.3)
- Moderate risk (9-16)	17	(10.6)
- High risk (≥17)	21	(13.1)
TMHI-15 (n = 156)		
Below average mental health (<43)	156	(100.0)
- <19	26	(16.7)
- 19-30	103	(66.0)
- 31-43	27	(17.3)

The results of multivariable regression analysis showed that GPAs below 3.0 (OR = 3.09, 95%CI: 1.17-8.14) and a family history of mental disorders (OR = 3.40, 95%CI: 1.10-10.48) were the independent predictors of moderate to severe mental health problems ( Tables 4 and 5).

**Table 4.** Factors associated with mental health problems (CGI).

Factors	Severity of mental health problems				P-value
	Normal to mild (n = 54)		Moderate to severe (n = 100)		
Gender					
Male	26	(48.1)	29	(29.0)	0.018
Female	28	(51.9)	71	(71.0)	
Age (years), Mean ± SD	21.41 ± 2.75		21.88 ± 3.27		0.368
Educational level					
Undergraduate	47	(87.0)	89	(89.0)	0.718
Postgraduate	7	(13.0)	11	(11.0)	
Programme					
Doctor of Medicine	33	(61.1)	57	(57.0)	0.858
Resident or Fellowship	6	(11.1)	11	(11.0)	
Others	15	(27.8)	32	(32.0)	
Course year (Doctor of Medicine n = 90)					
Preclinical year	20	(60.6)	40	(70.2)	0.353
Clinical year	13	(39.4)	17	(29.8)	
Admission route (Doctor of Medicine n = 90)					
The consortium of Thai Medical Schools	23	(69.7)	47	(82.5)	0.397
Quota	8	(24.2)	8	(14.0)	
Unknown	2	(6.1)	2	(3.5)	
GPA, Mean ± SD	3.28 ± 0.33		3.12 ± 0.45		0.370
<3.0	6	(11.1)	28	(28.0)	0.048
≥3.0	39	(72.2)	59	(59.0)	
Unspecified	9	(16.7)	13	(13.0)	
Medical underlying disease	9	(16.7)	25	(25.0)	0.517
Allergy	6	(11.1)	15	(15.0)	0.502
Asthma	2	(3.7)	2	(2.0)	0.612
Migraine	1	(1.9)	2	(2.0)	1.000
Others	1	(1.9)	6	(6.0)	0.422
A history of psychiatric treatment	9	(16.7)	15	(15.0)	0.506
A family history of mental disorders	4	(7.4)	22	(22.0)	0.030

Data are presented as number (%), mean ± standard deviation or median (interquartile range).

P-value corresponds to the Independent samples t-test, Mann-Whitney U test, Chi-square test or Fisher's exact test.

**Table 5.** Factors associated with mental health problems (CGI) analysed by univariable and multivariable regression analysis (n=154).

Factors	Univariable analysis			Multivariable analysis		
	OR ^1^	95%CI	P-value	OR _adj_ ^2^	95%CI	P-value
Sex						
Male	1.00	Reference		1.00	Reference	
Female	2.27	(1.14-4.52)	0.019	1.92	(0.94-3.94)	0.076
GPA						
<3.0	3.09	(1.17-8.14)	0.023	3.17	(1.18-8.56)	0.022
≥3.0	1.00	Reference		1.00	Reference	
A family history of mental disorder	3.40	(1.10-10.48)	0.033	3.78	(1.18-12.04)	0.025

Abbreviations: OR, Odds Ratio; ORadj, Adjusted Odds Ratio; CI, confident interval.

Variable was included in the multivariable model due to having a p-value < 0.050 in the univariable analysis.

^1^ Crude Odds Ratio estimated by Binary Logistic regression.

^2^ Adjusted Odds Ratio estimated by Multiple Logistic regression.

## Discussion

Mental health problems are a common issue in university students. The total number of Navamindradhiraj university students is approximately 2,500. In addition, around 260 students visited Suprara mental health clinic, so the point prevalence of mental health problems is around 10%. Moreover, this number is only for students who sought mental health treatment. Hence, the actual prevalence in a community-based setting is likely to be over 10%. The results revealed that medical students and residents of fellowship are the majority of participants in this study. We believe this is because students in the Faculty of Medicine, students are more familiar with the health service and have more exposure to the psychiatric unit due to their clinical coursework.

The most common psychiatric diagnoses in our study were depressive disorders, adjustment disorders and anxiety disorders, respectively. The prevalence of common mental disorders in this study was similar to the study by MacLean *et al.* [2016], Tung *et al.* [2018], and Pacheco *et al.* [2017]. Our study is among the first to present the prevalence of common mental health problems from a university mental health service. The prevalence of depression in the present study was higher than in prior studies [ Akhtar *et al.*, 2020; Pacheco *et al.*, 2017; Rotenstein *et al.*, 2016], likely because the participants were recruited from the mental health service. However, the anxiety rate was similar to earlier studies [ Phomprasith *et al.*, 2022]. Additionally, the three most common mental health problems presented in the service are similar to those in the community-based study of Thai university students [ Chiddaycha and Wainipitapong, 2021]. It can also be implied that university mental health service may benefit more from improving the service for depression and anxiety; for example, through the use of standard psychotherapy (cognitive behavioural therapy, interpersonal therapy) [ Hofmann *et al.*, 2012].

The PHQ-9 and 8Q questionnaires showed most participants had mild depression and a low risk of suicide. However, the overall CGI scale at baseline is four (moderately ill), which is clinically significant and needs to be tackled to prevent further complications and disease progression. All participants had below-average mental health from TMHI-15. However, TMHI-15 could be useful for monitoring treatment improvement in mental health status.

Interestingly, most clients rated our mental health service a very high satisfaction score. In addition, it can be seen that the median waiting time for the first psychiatric evaluation was zero days as the university policy intends to provide a standard of mental health care urgently and vitally for the students. Furthermore, more than half of our participants would like to use telemedicine. This is probably influenced by the COVID-19 pandemic. However, it can indicate that the clinic should implement telemedicine in the service. We will apply a full service of telemedicine to our clinic in the near future in order to increase accessibility to the service as well as avoid mental health stigma for our students. However, for high-risk cases, mental health teams should advise and convince them to come to evaluate further face-to-face.

The multivariable regression analysis demonstrated that having a low GPAs and a family history of mental disorders were strong predictors of moderate to severe mental health problems. Therefore, these factors may be helpful in screening mental health problems for university students.

There are several strengths in this study. First, the study was established in the specialised mental health service for university students, which is rare in Thailand. Secondly, we had planned to collect the data in all cases in our service, Finally, two authors extracted the diagnosis data following ICD-10 [ World Health Organization, 1992] separately in order to avoid missing data and bias.

We are aware of some limitations of this study. First, due to the cross-sectional descriptive design, we can only indicate associated factors, not causal relationships. Secondly, the results of the present came from Navamindradhiraj University, so it may have a different context from other universities, which may not be representative of all university students. Finally, most of the mental health screening questionnaires were self-rated scales; therefore, the assessment must be interpreted cautiously.

Future research should investigate the longitudinal data that can assess the effectiveness of our mental health service. Moreover, a study about mental health problems in university staff should be done because Supara mental health service also provides mental health consults to all university staff.

## Conclusions

The most common mental health problems in university students were depressive disorders. Low GPAs and a family history of mental disorder were independent predictors of moderate to severe mental health problems. Screening these factors may be helpful for the university to provide early detection and management.

## Data availability

### Underlying data

figshare: Data-Supara Mental Health Service. https://doi.org/10.6084/m9.figshare.21131953.v2 [ Chinvararak, 2022]

This project contains the following underlying data:

- Raw data-Pre-analysed.xlsx (Anonymised responses in excel sheet)

### Extended data

figshare: Data-Supara Mental Health Service. https://doi.org/10.6084/m9.figshare.21131953.v2 [ Chinvararak, 2022]

This project contains the following extended data:

- Data Record Form_Supara.docx (Blank English copy of the data record form used in this study)
- Figure 1.jpg

Data are available under the terms of the Creative Commons Attribution 4.0 International Lisence (CC BY 4.0).
