# Supplementary material for: Prevalence of common mental health problems and associated factors among university students visiting Supara mental health service: A cross-sectional study
Source: F1000Res. 2025 Apr 3;11:1107. Originally published 2022 Sep 27. [Version 5] doi: 10.12688/f1000research.126054.5 (PMC10122108; doi:10.12688/f1000research.126054.5)
Supplement: Peer review history

# Reviewer report: Reviewer response for version 5

**DOI:** 10.5256/f1000research.179772.r375432

**Published:** 2025-04-09

**Authors:** Thanapob Bumphenkiatikul

**Affiliations:** 1 Center of Excellence in Transgender Health (CETH), Faculty of Medicine, Chulalongkorn University, Bangkok, Thailand; 2 Division of Academics Affairs, Faculty of Medicine, Chulalongkorn University, Bangkok, Bangkok, Thailand

I approved the minor change made by the authors in the Acknowledgement section.

Is the work clearly and accurately presented and does it cite the current literature?

Yes

If applicable, is the statistical analysis and its interpretation appropriate?

Yes

Are all the source data underlying the results available to ensure full reproducibility?

Yes

Is the study design appropriate and is the work technically sound?

Yes

Are the conclusions drawn adequately supported by the results?

Yes

Are sufficient details of methods and analysis provided to allow replication by others?

Yes

Reviewer Expertise:

Transgender medicine, Medical education, Reproductive medicine

I confirm that I have read this submission and believe that I have an appropriate level of expertise to confirm that it is of an acceptable scientific standard.

# Reviewer report: Reviewer response for version 3

**DOI:** 10.5256/f1000research.146131.r168362

**Published:** 2023-04-21

**Authors:** Pracheth Raghuveer

**Affiliations:** 1 Department of Epidemiology, National Institute of Mental Health and Neurosciences, India, Bangalore, Karnataka, India

I am satisfied with the changes made by the authors. The manuscript may now be approved without reservations.

Is the work clearly and accurately presented and does it cite the current literature?

Yes

If applicable, is the statistical analysis and its interpretation appropriate?

Yes

Are all the source data underlying the results available to ensure full reproducibility?

Yes

Is the study design appropriate and is the work technically sound?

Yes

Are the conclusions drawn adequately supported by the results?

Yes

Are sufficient details of methods and analysis provided to allow replication by others?

Yes

Reviewer Expertise:

Maternal Mental Health, Non-Communicable Diseases, Geriatrics.

I confirm that I have read this submission and believe that I have an appropriate level of expertise to confirm that it is of an acceptable scientific standard.

# Reviewer report: Reviewer response for version 3

**DOI:** 10.5256/f1000research.146131.r168361

**Published:** 2023-04-05

**Authors:** Thanapob Bumphenkiatikul

**Affiliations:** 1 Center of Excellence in Transgender Health (CETH), Faculty of Medicine, Chulalongkorn University, Bangkok, Thailand; 2 Division of Academics Affairs, Faculty of Medicine, Chulalongkorn University, Bangkok, Bangkok, Thailand

After revision by the authors, the manuscript now includes an elaboration that satisfactory scores are assessed using a Likert-type scale ranging from 0 to 10 (0 = poor, 10 = excellent), which is a feasible measure for evaluating short-term outcomes of service provision. The service satisfaction score was rated at 9.45 ± 0.83, which could serve as an excellent key performance indicator (KPI) for the service provision. I am eagerly anticipating any subsequent reports that demonstrate the ultimate end-point of decreased prevalence of severe mental health problems among clients, which would indicate the long-term efficacy of the clinic.

Is the work clearly and accurately presented and does it cite the current literature?

Yes

If applicable, is the statistical analysis and its interpretation appropriate?

Yes

Are all the source data underlying the results available to ensure full reproducibility?

Yes

Is the study design appropriate and is the work technically sound?

Yes

Are the conclusions drawn adequately supported by the results?

Yes

Are sufficient details of methods and analysis provided to allow replication by others?

Yes

Reviewer Expertise:

Transgender Healthcare, Medical Education

I confirm that I have read this submission and believe that I have an appropriate level of expertise to confirm that it is of an acceptable scientific standard.

# Reviewer report: Reviewer response for version 1

**DOI:** 10.5256/f1000research.138429.r162187

**Published:** 2023-03-20

**Authors:** Pracheth Raghuveer

**Affiliations:** 1 Department of Epidemiology, National Institute of Mental Health and Neurosciences, India, Bangalore, Karnataka, India

The study highlights the prevalence and determinants of mental health problems among university students in a specialized mental health clinic. It is a well-written paper. There is further scope for improvement. Here are my constructive comments:

1. The authors mention that the majority of the participants were satisfied with the mental health services. But it is unclear how satisfaction was assessed. Was there a scale used? Please explain in the methods section.
2. The discussion needs major improvement. Please compare your findings with the findings of other studies. Speculate wherever necessary.
3. The strengths of this study or the novelty must be explained in the discussion.
4. Most of the assessments were done using self-rated scales. This must be mentioned as a limitation. 

Is the work clearly and accurately presented and does it cite the current literature?

Yes

If applicable, is the statistical analysis and its interpretation appropriate?

Yes

Are all the source data underlying the results available to ensure full reproducibility?

Yes

Is the study design appropriate and is the work technically sound?

Yes

Are the conclusions drawn adequately supported by the results?

Yes

Are sufficient details of methods and analysis provided to allow replication by others?

Yes

Reviewer Expertise:

Maternal Mental Health, Non-Communicable Diseases, Geriatrics.

I confirm that I have read this submission and believe that I have an appropriate level of expertise to confirm that it is of an acceptable scientific standard, however I have significant reservations, as outlined above.

# Response

**Published:** 2023-03-21

**Authors:** Chotiman Chinvararak

**Affiliations:** Faculty of Medicine Vajira Hospital, Navamindradhiraj University, Thailand

Thank you very much for your time and valuable comment in order to improve our manuscript. We really appreciate it. We have addressed all of your comments in the 2nd version of the manuscript.

 Sincerely yours

 Chotiman Chinvararak

 Coressponding author

# Reviewer report: Reviewer response for version 1

**DOI:** 10.5256/f1000research.138429.r152313

**Published:** 2022-11-09

**Authors:** Thanapob Bumphenkiatikul

**Affiliations:** 1 Center of Excellence in Transgender Health (CETH), Faculty of Medicine, Chulalongkorn University, Bangkok, Thailand; 2 Division of Academics Affairs, Faculty of Medicine, Chulalongkorn University, Bangkok, Bangkok, Thailand

The article describes common mental health problems and associated factors among university students visiting one in-house mental health clinic that provides services for students and staff of one university in Thailand. The results show depressive disorder, adjustment disorder, and anxiety disorder as the three most common psychiatric diagnoses among participants, which are similar to those in the community-based study in another Thai medical school. The study also highlights the association between lower grade point averages and a family history of mental disorders with moderate to severe mental health problems and suggests using these factors as a screening tool for mental health problems among university students. 

 The clinic was established and implemented in February 2020 to serve approximately 2,500 students and an additional number of all university staff. Despite caring for 260 students in 17 months, the waiting time for the first psychiatric evaluation was surprisingly reported as zero days. It would be more informative for readers if the authors mentioned the number of psychologists, mental health nurses, and psychiatrists providing care at the clinic, the total number of university staff and those seeking care at the clinic, the number of visits by several types of clients during the same 17 months period and statistics of the number of diagnoses given to each clients receiving the service. In the follow-up study, with an increasing number of clients, it would be interesting to investigate and compare the results between every subgroup, including undergraduate and postgraduate students, or medical and nursing students.

Is the work clearly and accurately presented and does it cite the current literature?

Yes

If applicable, is the statistical analysis and its interpretation appropriate?

Yes

Are all the source data underlying the results available to ensure full reproducibility?

Yes

Is the study design appropriate and is the work technically sound?

Yes

Are the conclusions drawn adequately supported by the results?

Yes

Are sufficient details of methods and analysis provided to allow replication by others?

Yes

Reviewer Expertise:

Transgender Healthcare, Medical Education

I confirm that I have read this submission and believe that I have an appropriate level of expertise to confirm that it is of an acceptable scientific standard.

# Response

**Published:** 2022-11-10

**Authors:** Chotiman Chinvararak

**Affiliations:** Faculty of Medicine Vajira Hospital, Navamindradhiraj University, Thailand

Thank you very much for your review and suggestions for our manuscript.

 In the follow-up study, which will have more participants, we plan to investigate the subgroup and make a comparison between undergraduate and postgraduate, between the faculty and the route of admission. We will design further study with the valuable recommendation from the reviewer.

 We appreciate your time reviewing, which assists in making the manuscript a better quality publication.

 Best regards 

 Chotiman Chinvararak
